# Patient perceptions and attitudes towards the use of artificial intelligence in the symptomatic breast unit

**DOI:** 10.1007/s00330-025-12288-4

**Published:** 2026-01-21

**Authors:** Sneha Singh, Rory Crean, Jessica O’Driscoll, Caitriona Cahir, Hayley Briody, Marie Bambrick, Neasa NiMhuircheartaigh, Niamh Hambly, Deirdre Duke, Maeve Mullooly, Nuala A Healy

**Affiliations:** 1 Department of Radiology, Royal College of Surgeons in Ireland, Dublin, Ireland; 2 Department of Surgery, Royal College of Surgeons in Ireland, Dublin, Ireland; 3 School of Population Health, RCSI University of Medicine and Health Sciences, Dublin, Ireland; 4 Beaumont Breast Centre, Beaumont Hospital, Dublin, Ireland

**Keywords:** Artificial intelligence, Breast imaging, Mammography, Deep learning, Patient survey

## Abstract

**Objectives:**

Artificial intelligence (AI) has been applied in a number of breast screening settings with favourable results. While there are a limited number of studies exploring patient attitudes on the use of AI in breast screening, none to date have examined patient perceptions on the use of AI in the symptomatic setting.

**Materials and methods:**

Following institutional approval, anonymous questionnaires were given to all patients attending the symptomatic breast clinic imaging department from 08/07/2024 to 04/10/2024. The questionnaire included questions on participant demographics and opinion questions on the use of AI in breast imaging. Multinomial logistic regression was performed to examine the associations between sociodemographic characteristics and patients' views about AI use in breast imaging.

**Results:**

One thousand five hundred thirty-four participants completed the questionnaire. Most participants were aged 40–59 years(35.8%). Almost one-quarter had either a personal (*n* = 372) or family history of breast cancer (*n* = 367). 61.4% (*n* = 943) had some/strong interest in AI. 46.3% (*n* = 711) agreed the use of AI in healthcare was a good idea, and 43.9% (*n* = 673) were indifferent. 61% (*n* = 935) agreed to a radiologist and AI tool reading their mammogram, and 66.9% (*n* = 1026) disagreed with AI being the sole reader of their mammogram. Even if AI was shown to be more accurate, 66.1% of patients still prefer a radiologist to review their mammogram, and even if AI was shown to be more efficient, 75.4% prefer a radiologist.

**Conclusion:**

Participants generally held favourable views towards the use of AI in healthcare. They welcome the use of AI as an adjunct for radiologists, but disagree with AI being the only reader of their mammogram.

**Key Points:**

***Question*** *Previous studies have explored patient attitudes on the use of AI in breast screening, but none to date have assessed this in the symptomatic setting*.

***Findings*** *The majority of participants welcome the use of AI as an adjunct for radiologists but disagree with AI being the sole reader of their mammogram*.

***Clinical relevance*** *This study highlights the importance of patient education to illustrate the benefits and limitations of AI in healthcare and how AI might work in the symptomatic breast setting*.

**Graphical Abstract:**

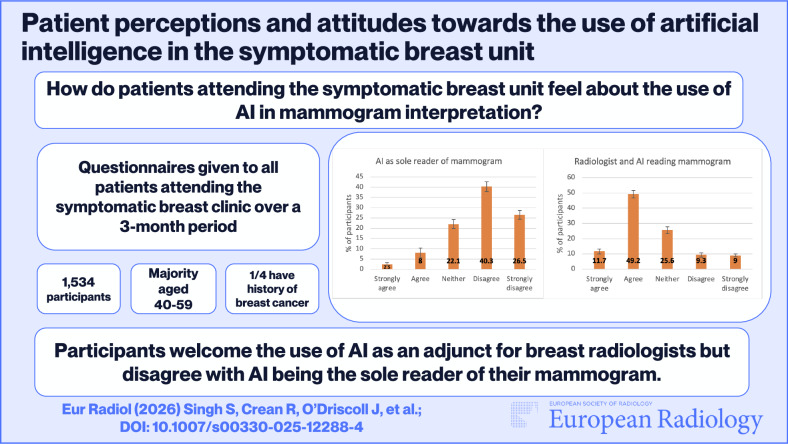

## Introduction

Studies have demonstrated that the incorporation of interpretative AI software into breast screening programmes can deliver similar or improved cancer detection rates when compared with breast radiologists [1, 2]. In addition to improved cancer detection rates, adoption of AI tools can also lead to reduced workload and increased efficiency in the context of breast screening [2–4]. To date, most of the initial studies on AI in breast imaging have focused on screening [5–12], with the attention now turning to its role as a clinical decision support in symptomatic breast imaging [13]. Non-interpretive AI tools can also be used in the symptomatic unit to improve workflow, and, ultimately, efficiency in the breast imaging department by triaging imaging requests [14] and extracting information from patient reports [15].

The introduction of AI in radiology has been mostly welcomed by radiology staff, with one particular survey study reporting that respondents feel breast imaging will be the radiology subspecialty most impacted by AI [16]. Furthermore, a dedicated study of breast imaging staff found that the majority of breast radiologists feel that the use of AI in screening would improve workflow [17]. However, prior to the implementation of AI in the symptomatic breast services, it is pertinent to assess and understand patients’ views and opinions on AI, to determine acceptability. The Irish National Breast Screening Programme offers free screening to all women 50–69 years of age with a mammogram every two years. These screening mammograms are read by two consultant radiologists, while mammograms in the symptomatic setting are usually read by one consultant, as is the case in most centres in the UK and Europe. While the idea of incorporating AI in healthcare is an exciting subject for many healthcare workers and software developers, it is anticipated that some patients may be apprehensive of its use in their own care.

There are a limited number of studies in the literature evaluating patient perceptions of AI in breast imaging, with previous research focused on asymptomatic patients attending for breast screening [5–7, 10]. There have been no published studies to date assessing patient opinions on the use of AI in the symptomatic breast clinic setting. This cohort represents a different group of patients from those attending for screening. They may have increased anxiety, been referred for investigation of breast symptoms, have a personal history or family history of breast cancer or be attending a clinic for breast procedures. As the role of diagnostic AI expands into the symptomatic setting, the aim of this study is to evaluate perceptions and opinions on the use of AI in breast imaging among patients attending a symptomatic breast unit in Ireland.

## Methods

Approval for this study was obtained from the local audit committee in our institution (audit number CA2024/126).

This was an observational, cross-sectional study in which patient perceptions on the use of AI in the symptomatic breast clinic were evaluated using a voluntary, fully anonymised questionnaire, devised by the authors of this study based on the literature review conducted. The paper-based survey consisted of 15 tick-box questions assessing patient demographics and opinions on specific AI applications in breast imaging using a Likert scale ranging from 1 = strongly agree to 5 = strongly disagree (Supp. Fig. 1). For the demographic questions, such as ethnicity, we used categories taken from the national inpatient survey of Ireland [18]. To measure the participants’ level of education, we used categories taken from the Irish education system because these were easiest to understand for respondents. The junior certificate is a board exam completed halfway through second-level education (post-primary), and the leaving certificate is completed at the end of second-level education. A Post Leaving Certificate (PLC) course is done post-second-level education.

The questionnaire was not pilot tested. The survey was designed by the authors following a review of studies published in the screening setting of patient perceptions on AI. To expand on the theme and learn more about patients’ thoughts in a diagnostic setting, some novel questions were developed. The questionnaire was circulated among staff in the department, including four breast radiology consultants, breast radiographers and two breast secretaries who distributed the questionnaires. These staff members reviewed the questionnaire and provided feedback, which was implemented into the questionnaire prior to starting the study. Patients attending the imaging department of the symptomatic breast clinic at our institution from the 8th of July 2024 to the 4th of October 2024 were invited to complete the voluntary paper-based survey while waiting for their scan. All questionnaires returned were collected and analysed. Questionnaires were excluded from the final sample if the following exclusion criteria were met; no AI questions answered at all, more than four AI questions not answered, and no demographic questions answered. The total number of AI questions in the questionnaire was eight, hence the threshold of exclusion of four AI questions was chosen as this represents half of these questions.

### Statistical analysis

Descriptive statistics (proportions [%]) were used to summarise the demographic characteristics of the analytical sample (participants with completed questionnaires) and to evaluate participants’ opinions and perceptions on the use of AI in breast imaging.

Four separate multinomial logistic regression models were performed to examine the associations between participant sociodemographic characteristics (age, education level) and responses to the following 4 questions about AI use in breast imaging; (i) how participants feel about an AI tool reading their mammograms along with a radiologist, (ii) how participants feel about an AI tool only reading their mammograms, (iii) whether participants prefer AI to a radiologist if AI were more accurate and (iv) more efficient. The five-point Likert scales were collapsed into three categories: ‘agree’, ‘neither agree nor disagree’ and ‘disagree’, to facilitate interpretation and to establish any differences in response categories by age and education level. Women aged 50–69 were included in one age band, as this represents the breast screening age group in Ireland. Neither agree nor disagree (labelled as ‘neutral response’ in the text) was used as the reference (baseline) category. The four multinomial regression models were adjusted for a family history of breast cancer, a previous breast cancer diagnosis and an interest in AI. Results are reported as relative risk ratios (RRR) with the corresponding 95% confidence interval (CI). The level of statistical significance was considered *p* < 0.05 for all statistical tests. Data analysis was performed using Stata SE 18.5 software.

## Results

### Characteristics of the study population

**Fig. 1:**
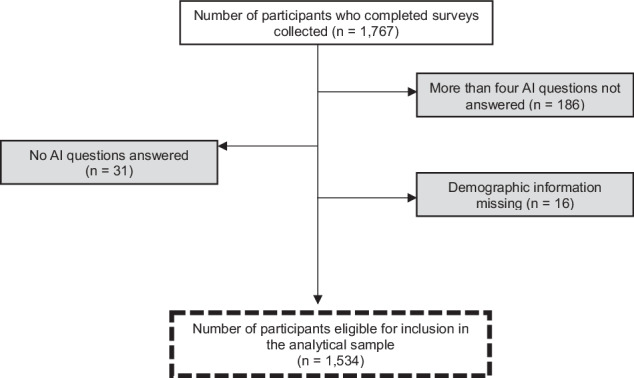
Flow chart of the study sample with reasons for exclusion in the grey boxes and the subsequent analytical samples in the dashed boxes

A total of 1767 surveys were completed over the 3-month study period, in which 233 responses were excluded, resulting in a total number of 1534 questionnaires eligible for inclusion in the analytical sample (Fig. 1). The demographic characteristics of participants are outlined in Table 1. In terms of age, the majority of participants were aged 40–59, with 549 respondents (35.8%) aged 40–49 years and 402 respondents (26.2%) aged 50–59 years. A total of 411 respondents (26.8%) held a Bachelor’s degree or higher. Almost a quarter of the participants had a first-degree relative with breast cancer or a personal history of breast cancer.

**Table 1:** Demographics of respondents

	Final analytical sample (*n* = 1534)	
Age (years)	*N* (%)	
< 30	50 (3.3%)	
30–39	233 (15.2%)	
40–49	549 (35.8%)	
50–59	402 (26.2%)	
60–69	163 (10.6%)	
≥ 70	135 (8.8%)	
Unknown	2 (0.1%)	
Ethnicity	*N* (%)	Population benchmark (22)
White Irish	1267 (82.6%)	76.6%
Irish traveller	6 (0.4%)	0.6%
Any other white background	166 (10.8%)	9.9%
Black, African	26 (1.7%)	1.3%
Any other black background	-	0.2%
Asian, Chinese	27 (1.8%)	0.5%
Any other Asian background	20 (1.3%)	1.9%
Other ethnic background	12 (0.8%)	1.3%
Prefer not to say	6 (0.4%)	
Unknown	4 (0.3%)	
Education	*N* (%)	
Junior certificate or below	184 (12.0%)	
Leaving certificate or equivalent	386 (25.2%)	
PLC course	224 (14.6%)	
Bachelor’s degree	411 (26.8%)	
Master’s or higher degree	270 (17.6%)	
Unknown	59 (3.8%)	
Previous breast cancer diagnosis	*N* (%)	
Yes	372 (24.3%)	
No	1154 (75.2%)	
Unknown	8 (0.5%)	
Family history of breast cancer (parent/sibling)	*N* (%)	
Yes	367 (23.9%)	
No	1145 (74.6%)	
Unknown	22 (1.4%)	
Reason for breast clinic attendance	*N* (%)	
Investigation of a breast symptom	770 (50.2%)	
Mammogram for a history of breast cancer	294 (19.2%)	
Mammogram for family history	201 (13.1%)	
Breast biopsy	7 (0.5%)	
Clinic follow-up	236 (15.4%)	
Other	-	
Unknown	26 (1.7%)	

Reasons for attending the breast clinic, included investigation of a breast symptom (*n* = 770, 50.2%), with the remaining attending for other reasons, such as for a mammogram for a personal history of breast cancer (19.2%), or a follow-up clinic visit (15.4%). A total of 732 participants (47.7%) had some interest in AI, with 211 participants (14%) declaring a strong interest and 331 (22%) declaring no interest. When asked more specifically if participants felt the use of AI in healthcare was a good idea, 711 participants agreed (46.3%), with only 113 (7.4%) disagreeing and the remaining 46% having a neutral response/no response to this question. Overall, the majority of participants expressed an interest in AI; however, the responses were equivocal when asked if the use of AI in healthcare is a good idea.

### Perceptions and opinions on the use of AI in breast imaging

**Fig. 2:**
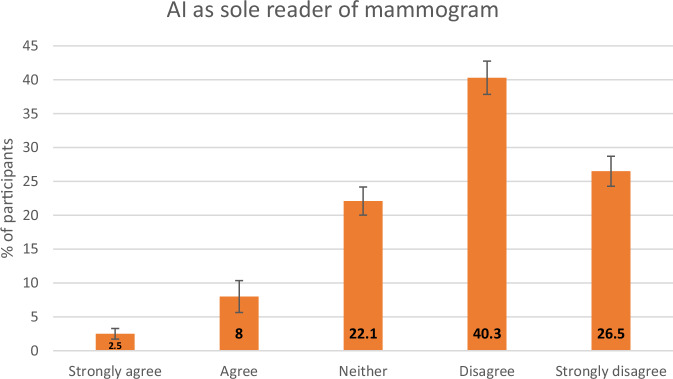
Responses to a question asking participants their opinion on AI being the only reader of their mammogram. The *y*-axis represents the percentage of participants, with the error bars representing 95% CIs

**Fig. 3:**
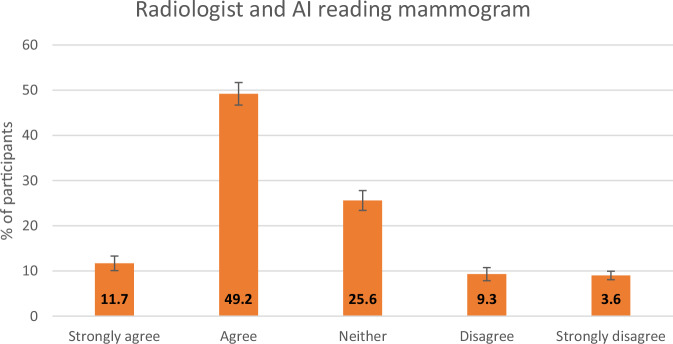
Responses to a question asking participants their opinion on AI and a radiologist reading their mammogram. The *y*-axis represents the percentage of participants, with the error bars representing 95% CIs

**Fig. 4:**
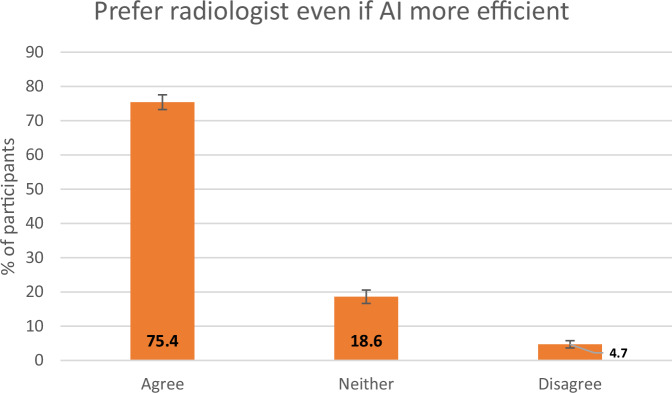
Responses to a question asking participants if they prefer a radiologist even if AI is more efficient. The *y*-axis represents the percentage of participants, with the error bars representing 95% CIs

**Fig. 5:**
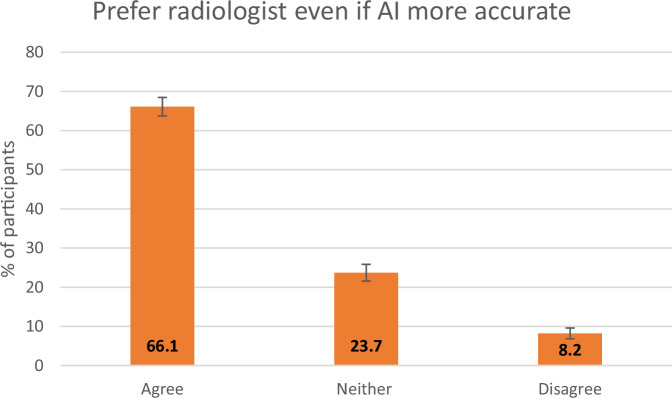
Responses to a question asking participants if they prefer a radiologist even if AI was shown to be more accurate. The *y*-axis represents the percentage of participants, with the error bars representing 95% CIs

Most participants (66.9%, *n* = 1026) disagreed with an AI tool being the sole reader of their mammogram in the future with only a minority (10.4%, *n* = 160) agreeing with this (Fig. 2). The majority of participants (61%, *n* = 935) agreed to their mammogram being read by both a radiologist and an AI tool in the future, while 12.9% (*n* = 198) disagreed with this and 25.5% (*n* = 392) neither agreed nor disagreed (Fig. 3). As a follow-on question, 75.4% (*n* = 1156) of respondents preferred to have a radiologist report their mammogram even if an AI tool were more efficient, (Fig. 4.) while 66.1% (*n* = 1014) of respondents preferred a radiologist even if AI were more accurate (Fig. 5.).

**Fig. 6:**
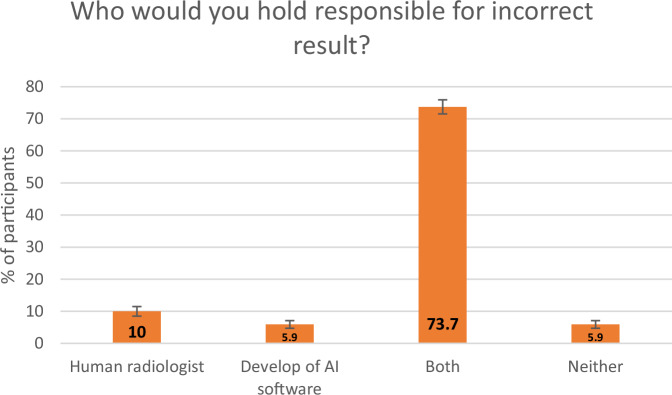
Responses to a question asking participants who they would hold responsible for an incorrect mammogram result when the mammogram is read by both a radiologist and an AI tool. The *y*-axis represents the percentage of participants, with the error bars representing 95% CIs

If interpretation errors were made when both an AI tool and a radiologist were interpreting a mammogram, most respondents (73.7%, *n* = 1130) would hold both the reporting radiologist and the software developer accountable for this error (Fig. 6). Only 350 respondents (22.8%) believe that breast radiologists will be replaced by AI in the future, however nearly one-third (31%) were unsure (Supp Fig. 2). The majority of participants (70.6%, *n* = 1083) agreed to their anonymised images being used for future research on AI.

### Multinomial regression analysis: associations between participant sociodemographics and perceptions and opinions on AI use in breast cancer imaging

In terms of age, the expected risk of agreeing with AI and a radiologist reading the mammogram compared to a neutral response was 1.55 times higher for participants in the 50-69 years age group compared to those in the < 40 years age group (adjusted RRR 1.55, 95% CI: 1.04–2.32, *p* = 0.03) (Table 2) The expected risk of disagreeing with AI as a sole reader of mammogram compared to a neutral response was significantly higher in those aged 40–49 years (adjusted RRR 1.50, 95% CI: 1.03–2.16, *p*-0.03) and in those aged 50–69 years (adjusted RRR 1.59, 95% CI: 1.08–2.36, *p* = 0.02) compared to those aged < 40 years (Table 3). The expected risk of preferring a radiologist even if the AI tool was more efficient compared to a neutral response was also significantly higher in those aged 50–69 years compared to those aged less than 40 years (adjusted RRR 1.52, 95% CI: 1.02–2.26, *p* = 0.04) (Table 4). The expected risk of preferring a radiologist even if the AI tool were more accurate was significantly higher in those aged 50–69 years and those aged 70 years compared to those aged < 40 years (50–69 years: adjusted RRR 1.60, 95% CI: 1.12–2.32, *p* = 0.01. > 70 years: adjusted RRR 1.84, 95% CI: 1.03–3.28, *p* = 0.04) (Supp Table 1).

**Table 2:** Multinomial logistic regression on the question asking participants if they agree with AI and a radiologist reading their mammogram

AI and a radiologist reading a mammogram—agree	Relative risk (RRR)	*p* value	95% CI
Age			
< 40 years	1 (base)		
40–49 years	1.32	0.145	0.91–1.92
50–69 years	1.55	0.032	1.04–2.32
> 70 years	1.77	0.052	0.99–3.16
Education level			
Leaving certificate and below	1 (base)		
PLC/bachelors and above	2.33	< 0.001	1.75–3.12
History of breast cancer			
Family history of breast cancer	0.89	0.476	0.65–1.22
Personal history of breast cancer	1.35	0.085	0.96–1.91
Interest in AI			
No interest	1 (base)		
Some interest	2.76	< 0.001	1.97–3.85
Strong interest	10.81	< 0.001	5.76–20.27
Don’t know	0.62	< 0.001	0.41–0.93
**Neither agree nor disagree**	**Base outcome**		
**AI and a radiologist reading a mammogram—disagree**	**Relative risk (RRR)**	***p*** **value**	**95% CI**
Age			
< 40 years	1 (base)		
40–49 years	1.09	0.736	0.66–1.79
50–69 years	1.29	0.346	0.76–2.20
> 70 years	0.65	0.324	0.28–1.53
Education level			
Leaving certificate and below	1 (base)		
PLC/bachelors and above	1.50	0.043	1.01–2.21
History of breast cancer			
Family history of breast cancer	0.83	0.397	0.54–1.28
Personal history of breast cancer	1.13	0.616	0.70–1.81
Interest in AI			
No interest	1 (base)		
Some interest	0.44	< 0.001	0.29–0.68
Strong interest	0.51	0.194	0.19–1.41
Don’t know	0.41	< 0.001	0.25–0.66

**Table 3:** Multinomial logistic regression on the question asking participants if they agree with AI being the sole reader of their mammogram

AI as sole reader of mammogram—agree	Relative Risk (RRR)	*p* value	95% CI
Age			
< 40 years	1 (base)		
40–49 years	1.22	0.682	0.65–1.95
50–69 years	1.15	0.629	0.65–2.06
> 70 years	0.88	0.768	0.37–2.07
Education level			
Leaving certificate and below	1 (base)		
PLC/Bachelors and above	1.22	0.377	0.79–1.88
History of breast cancer			
Family history of breast cancer	0.79	0.363	0.48-1.31
Personal history of breast cancer	0.77	0.335	0.45-1.32
Interest in AI			
No interest	1 (base)		
Some interest	3.32	0.002	1.54–7.14
Strong interest	9.24	< 0.001	4.10–20.80
Don’t know	2.13	0.082	0.91–4.99
**Neither agree nor disagree**	**Base outcome**		
**AI as sole reader of mammogram—disagree**	**Relative risk (RRR)**	***p*** **value**	**95% CI**
Age			
< 40 years	1 (base)		
40–49 years	1.50	0.033	1.03–2.16
50–69 years	1.59	0.019	1.08–2.36
> 70 years	0.87	0.597	0.51–1.48
Education			
Leaving certificate and below	1 (base)		
PLC/bachelors and above	2.10	< 0.001	1.58–2.80
History of breast cancer			
Family history of breast cancer	1.00	0.958	0.74–1.38
Personal history of breast cancer	1.38	0.062	0.98–1.93
Interest in AI			
No interest	1 (base)		
Some interest	0.96	0.802	0.68–1.35
Strong interest	0.56	0.014	0.35–0.89
Don’t know	0.52	0.001	0.34–0.78

**Table 4:** Multinomial logistic regression on the question asking participants if they prefer a radiologist, even if AI is more efficient

Prefer a radiologist even if AI is more efficient – agree	Relative Risk (RRR)	*p* value	95% CI
Age			
< 40 years	1 (base)		
40–49 years	1.31	0.155	0.90–1.90
50–69 years	1.52	0.041	1.02–2.26
> 70 years	1.25	0.463	0.69–2.24
Education level			
Leaving certificate and below	1 (base)		
PLC/Bachelors and above	1.20	0.223	0.89–1.62
History of breast cancer			
Family history of breast cancer	1.12	0.501	0.80–1.56
Personal history of breast cancer	1.09	0.641	0.77–1.54
Interest in AI			
No interest	1 (base)		
Some interest	0.83	0.319	0.57–1.20
Strong interest	0.77	0.295	0.48–1.25
Don’t know	0.63	0.045	0.50–0.99
**Neither agree nor disagree**	**Base outcome**		
**Prefer a radiologist even if AI is more efficient—disagree**	**Relative risk (RRR)**	***p*** **value**	**95% CI**
Age			
< 40 years	1 (base)		
40–49 years	0.66	0.277	0.32–1.39
50–69 years	0.82	0.623	0.38–1.79
> 70 years	0.28	0.123	0.06–1.41
Education level			
Leaving certificate and below	1 (base)		
PLC/bachelors and above	2.07	0.029	1.08–3.99
History of breast cancer			
Family history of breast cancer	1.66	0.114	0.89–3.09
Personal history of breast cancer	2.00	0.038	1.03–3.86
Interest in AI			
No interest	1 (base)		
Some interest	1.22	0.652	0.52–2.88
Strong interest	2.46	0.065	0.94–6.39
Don’t know	1.57	0.377	0.58–4.30

With regards to education level, the expected risk of both agreeing and disagreeing with a radiologist and AI tool reading their mammogram compared to a neutral response was significantly higher in participants who held a Bachelor’s degree and above compared to those with second-level education and below (adjusted RRR 2.33, 95% CI: 1.75-3.12, *p* < 0.001 agree; adjusted RRR 1.50 95% CI: 1.01–1.21 disagree, *p* = 0.04) (Table 2) The expected risk of disagreeing with AI as a sole reader of mammogram was significantly higher in those with Bachelor’s degree and above compared to those with second-level education and below (adjusted RRR 2.10, 95% CI: 1.58–2.80, *p* < 0.001 (Table 3). The expected risk of disagreeing with preferring a radiologist even if AI were more efficient (adjusted RRR 2.07, 95% CI: 1.08–3.99, *p* = 0.03) and disagreeing with preferring a radiologist even if AI more accurate (adjusted RRR 3.25, 95% CI: 1.84–5.76, *p* < 0.001) compared to a neutral response was significantly higher in those with third level education compared to those without (Table 4 and Supp Table 1).

The expected risk of both agreeing and disagreeing with an AI tool and radiologist reading their mammogram compared to not agreeing or disagreeing was significantly higher in participants with some interest and strong interest in compared to those with no interest in AI (Table 2). While the expected risk of agreeing with AI as a sole reader of mammogram compared to a neutral response was significantly higher in participants with some or strong interest in AI, the expected risk of disagreeing was significantly lower in those with strong interest in AI and neither (Table 3). The expected risk of agreeing with preferring a radiologist, even if AI were more efficient compared to a neutral response, was significantly lower for participants who were unsure about their interest in AI (Table 4). While the expected risk of disagreeing with preferring a radiologist, even if AI were more accurate compared to a neutral response, was significantly higher for participants with a strong interest in AI (Supp. Table 1).

## Discussion

This study utilised a voluntary, anonymous questionnaire to assess patient perceptions of the use of AI in breast imaging in the symptomatic breast setting. We identified that 61% of participants agree with the use of an AI tool in combination with a radiologist for mammogram interpretation. Two-thirds of patients disagree with AI being the sole reader of their mammogram, and in most instances would prefer radiologist involvement even if the AI tool is more efficient and more accurate. Respondents aged 50–69, those with higher levels of education and those with greater interest in AI were noted to have more favourable views of AI in breast imaging.

Overall, women in our study population have a positive view of AI in breast imaging, with findings comparable to previous studies on this topic. A study of women aged 16–75 years in the Dutch population found that 45.9% agreed with AI as a second reader in screening mammography [11]. In a study conducted on female NHS workers and their friends or family [6], 47.2% of women were positive about the use of AI in breast screening. Our results are similar to a study conducted on women attending breast screening in Norway, where 64% were willing to participate in a study where AI was being used to read mammograms along with radiologists [7]. A larger proportion of women in our study were agreeable to AI being used in image interpretation compared to the UK and Dutch studies, and it can be hypothesised that this could be due to the population being a symptomatic breast patient group, in which half the participants were attending due to follow-up mammograms and breast procedures, etc. These patients may have more awareness of breast imaging compared to the general public and have prior experience undergoing mammograms/breast cancer treatment.

Multinomial logistic regression in this study showed that the expected risk of agreeing compared to having a neutral response to double reading by a radiologist and AI software was higher for participants in the screening age group (50–69 years), which is similar to the trends reported by Lennox-Chhugani et al [6]. Women who reported some or a strong baseline personal interest in AI were more likely to agree to double reading by a radiologist and AI, which was also demonstrated by Holen et al [7]. Similar to prior studies [10, 12], we found that the expected risk of agreeing with AI being used in image interpretation compared to having a neutral response was higher in participants with third-level education. However, we also found the expected risk of disagreeing with AI being the sole reader of a mammogram to be higher in participants with a third-level education compared to the neutral response. It can be hypothesised that women with higher levels of education may have more background knowledge of AI and therefore be more agreeable to its introduction, but cautious about its autonomous use in imaging.

Collectively, this current study has highlighted the importance of educating patients about the use of AI in breast imaging. When asked about their general interest in AI, 22% of participants responded with ‘no interest’ and 15% selected the option ‘don’t know’. Among the participants, 44% were unsure if the use of AI in healthcare was a good idea, while 7% disagreed with this statement. These results are similar to a study by Holen et al, which showed that 46.8% of participants had no or little knowledge of AI in healthcare [7]. A qualitative study by Carter et al using discussion groups revealed that women would like to be made aware of research conducted on AI in screening mammography interpretation, would like long testing periods before the full implementation of AI and strong cross-checking protocols by radiologists [5]. Conducting a patient questionnaire before and after a short education programme on AI would be interesting to determine if responses changed.

Although AI has multiple benefits in radiology, there are some associated limitations. Research has shown that when AI generates incorrect reports or findings, radiologists make more errors than they would have without AI [19]. In our study, 74% of participants would hold both the radiologist and the software developer of the AI tool responsible for a reporting error when both parties are involved in image interpretation. This figure is greater than a study conducted by Pesapane et al, who reported that 52% of respondents would hold both the radiologist and software developer accountable for the error [12]. In our study, 10% of respondents would blame the radiologist only, which is less than the 38.7% respondents identified by a study from Ongena et al [11]. The current lack of clarity on the regulation and governance of AI in breast imaging remains a concern for patients [6] and clinicians alike, and is the most pressing issue that should be addressed prior to AI implementation in breast imaging. Of note, a discussion group study by Johansson et al interestingly found that patients had a much higher tolerance for mistakes made by radiologists than by AI [9], and this was also supported by a study published in 2024 by Carter et al [20]. Participants in that study expressed that if AI were to make a mistake, it would be the responsibility of clinicians, as AI is not capable of being held accountable for its errors [9]. The findings in the current study suggest that further education for patients around the use of AI in healthcare, and in particular breast imaging, will be important, to ensure acceptability and understanding of how AI might work in the routine symptomatic breast clinic setting. Information leaflets with links to online education websites could be provided to patients prior to their mammograms, so they have a better understanding of AI in breast imaging when they attend for their scans and consent to AI being used for interpretation.

Limitations of this current study include that the survey was only available in English, and therefore, non-English speakers are likely underrepresented. Furthermore, as the questions were tick-box only, participants did not have the opportunity to express their opinions in free text. While the study includes a large sample size, respondents expressed lower levels of third-level education compared to the Irish population, as per figures published by the Central Statistics Office (CSO) [21], and the number of participants who identified as White Irish ethnicity was higher than the Irish population [22]. Finally, as the study ran for a four-month period, it may not have captured the opinions of this study group extensively, and further studies should be conducted over a longer period. With regards to response rate, although the authors perceive a high response rate, data on the number of patients attending the breast unit was not recorded, and therefore not possible to calculate the actual response rate.

In conclusion, this study demonstrates that patients attending the symptomatic breast unit generally have a favourable view towards the use of AI in healthcare and most welcome the use of AI as an aid for radiologists in mammography interpretation in the symptomatic breast clinic setting. Screening-aged women, women with higher education levels and greater interest in AI, expressed more positive attitudes to AI in combination with a radiologist. Respondents were not comfortable with the idea of autonomous AI, even if AI is considered more accurate and efficient than radiologists. This study highlights the importance of patient education to illustrate the benefits and limitations of AI in healthcare and how AI might work in the symptomatic breast setting. The findings of this study are valuable and encouraging for both breast radiologists and software developers of AI algorithms. Our findings may aid clinicians in the future to adopt a tailored consent process when utilising AI in image interpretation. Patients can specify to what extent they would consent to AI being applied in their care. Patients are at the epicentre of what we do in breast imaging, and it is essential that we take their views into consideration particularly when adopting AI into the symptomatic breast unit or when embarking on future studies of AI in this setting.

### Data sharing

No identifiable patient data was collected in this study. The questionnaires were anonymous.

## Supplementary information

ELECTRONIC SUPPLEMENTARY MATERIAL

## References

[1] Dembrower K, Crippa A, Colón E, Eklund M, Strand F (2023) Artificial intelligence for breast cancer detection in screening mammography in Sweden: a prospective, population-based, paired-reader, non-inferiority study. Lancet Digit Health 5:e703–e711 37690911 10.1016/S2589-7500(23)00153-X

[2] Lång K, Josefsson V, Larsson AM et al (2023) Artificial intelligence-supported screen reading versus standard double reading in the Mammography Screening with Artificial Intelligence trial (MASAI): a clinical safety analysis of a randomised, controlled, non-inferiority, single-blinded, screening accuracy study. Lancet Oncol 24:936–944 37541274 10.1016/S1470-2045(23)00298-X

[3] van Winkel SL, Rodríguez-Ruiz A, Appelman L et al (2021) Impact of artificial intelligence support on accuracy and reading time in breast tomosynthesis image interpretation: a multi-reader multi-case study. Eur Radiol 31:8682–8691 33948701 10.1007/s00330-021-07992-w PMC8523448

[4] Rodríguez-Ruiz A, Krupinski E, Mordang JJ et al (2019) Detection of breast cancer with mammography: effect of an artificial intelligence support system. Radiology 290:305–314 30457482 10.1148/radiol.2018181371

[5] Carter SM, Carolan L, Saint James Aquino Y et al (2023) Australian women’s judgements about using artificial intelligence to read mammograms in breast cancer screening. Digit Health 9:20552076231191057 37559826 10.1177/20552076231191057 PMC10408316

[6] Lennox-Chhugani N, Chen Y, Pearson V, Trzcinski B, James J (2021) Women’s attitudes to the use of AI image readers: a case study from a national breast screening programme. BMJ Health Care Inform 28:e100293 10.1136/bmjhci-2020-100293 PMC8021737 33795236

[7] Holen ÅS, Martiniussen MA, Bergan MB, Moshina N, Hovda T, Hofvind S (2024) Women’s attitudes and perspectives on the use of artificial intelligence in the assessment of screening mammograms. Eur J Radiol 175:111431 38520804 10.1016/j.ejrad.2024.111431

[8] de Vries CF, Morrissey BE, Duggan D, Staff RT, Lip G (2021) Screening participants’ attitudes to the introduction of artificial intelligence in breast screening. J Med Screen 28:221–222 33715512 10.1177/09691413211001405

[9] Viberg Johansson J, Dembrower K, Strand F, Grauman Å (2024) Women’s perceptions and attitudes towards the use of AI in mammography in Sweden: a qualitative interview study. BMJ Open 14:e084014 38355190 10.1136/bmjopen-2024-084014 PMC10868248

[10] Jonmarker O, Strand F, Brandberg Y, Lindholm P (2019) The future of breast cancer screening: What do participants in a breast cancer screening program think about automation using artificial intelligence?. Acta Radiol Open 8:2058460119880315 31839989 10.1177/2058460119880315 PMC6901736

[11] Ongena YP, Yakar D, Haan M, Kwee TC (2021) Artificial intelligence in screening mammography: a population survey of women’s preferences. J Am Coll Radiol 18:79–86 33058789 10.1016/j.jacr.2020.09.042

[12] Pesapane F, Rotili A, Valconi E et al (2023) Women’s perceptions and attitudes to the use of AI in breast cancer screening: a survey in a cancer referral centre. Br J Radiol 96:20220569 36314388 10.1259/bjr.20220569 PMC11864346

[13] Royal Marsden NHS Foundation Trust (2024) Use of Artificial Intelligence in the Symptomatic BReAst Clinic SEtting. Active clinical trial (ID: NCT06578988) available at: https://clinicaltrials.gov/study/NCT06578988

[14] Yala A, Lehman C, Schuster T, Portnoi T, Barzilay R (2019) A deep learning mammography-based model for improved breast cancer risk prediction. Radiology 292:60–66 31063083 10.1148/radiol.2019182716

[15] Sippo DA, Warden GI, Andriole KP et al (2013) Automated extraction of BI-RADS final assessment categories from radiology reports with natural language processing. J Digit Imaging 26:989–994 23868515 10.1007/s10278-013-9616-5 PMC3782591

[16] Al Mohammad B, Aldaradkeh A, Gharaibeh M, Reed W (2024) Assessing radiologists’ and radiographers’ perceptions on artificial intelligence integration: opportunities and challenges. Br J Radiol 97:763–769 38273675 10.1093/bjr/tqae022 PMC11027289

[17] Tang JS, Frazer HM, Kunicki K, Basnayake P, Omori M, Lippey J (2024) Australian healthcare workers’ views on artificial intelligence in BreastScreen: results of a mixed method survey study. Prev Med Rep 48:102917 39558908 10.1016/j.pmedr.2024.102917 PMC11570924

[18] Programme NCE (2024) Findings of the 2024 National Inpatient Experience Survey. Technical report https://yourexperience.ie/wp-content/uploads/2025/05/National-Inpatient-Experience-Survey_Technical_Report_2024_Final.pdf

[19] Bernstein MH, Atalay MK, Dibble EH et al (2023) Can incorrect artificial intelligence (AI) results impact radiologists, and if so, what can we do about it? A multi-reader pilot study of lung cancer detection with chest radiography. Eur Radiol 33:8263–8269 37266657 10.1007/s00330-023-09747-1 PMC10235827

[20] Carter SM, Popic D, Marinovich ML, Carolan L, Houssami N (2024) Women’s views on using artificial intelligence in breast cancer screening: a review and qualitative study to guide breast screening services. Breast 77:103783 39111200 10.1016/j.breast.2024.103783 PMC11362777

[21] CSO. 2021. Educational attainment thematic report 2022 Ireland. Central Statistics Office

[22] CSO (2022) Census of population 2022 profile 5—diversity, migration, ethnicity, irish travellers & religion Ireland. Available via https://www.cso.ie/en/releasesandpublications/ep/p-cpp5/censusofpopulation2022profile5-diversitymigrationethnicityirishtravellersreligion/keyfindings/

