# Supplementary material for: Patient perceptions and attitudes towards the use of artificial intelligence in the symptomatic breast unit
Source: Eur Radiol. 2026 Jan 21;36(6):4467–77. doi: 10.1007/s00330-025-12288-4 (PMC13212389; doi:10.1007/s00330-025-12288-4)
Supplement: Supplementary file 1 — ELECTRONIC SUPPLEMENTARY MATERIAL [file 330_2025_12288_MOESM1_ESM.pdf]

Patient Perceptions and Attitudes Towards the use of Artificial Intelligence in the Symptomatic Breast Unit

ELECTRONIC SUPPLEMENTARY MATERIAL

Eur Radiol (2025) Singh S, Crean R, O’Driscoll J, et al.

Questionnaire:

Patient perceptions of the use of Artificial Intelligence (AI) in the symptomatic breast unit

Artificial Intelligence (AI) tools are showing huge promise in assisting doctors who report imaging (radiologists), to improve workflow and reporting, and help doctors to identify areas of concern on mammogram.

*This is an anonymous, voluntary questionnaire we are conducting to understand patient's opinions on the use of AI in healthcare, particularly in breast imaging. We currently do **not** use any AI tools for reporting mammograms in the Beaumont Breast Centre.*

1. What age are you?

15 – 29 years ☐ 50 – 59 years ☐
30 – 39 years ☐ 60 – 69 years ☐
40 – 49 years ☐ >70 years ☐

2. What is your ethnicity?

White Irish ☐ Any other black background ☐ Prefer not to say ☐
Irish traveller ☐ Asian, Chinese ☐
Any other white background ☐ Any other Asian background ☐
Black, African ☐ Other Ethnic background ☐

3. What is your highest level of education?

Junior certificate or equivalent ☐ Bachelor's degree ☐
Leaving certificate or equivalent ☐ Masters or higher degree ☐
PLC Course ☐

4. Have you ever been diagnosed with breast cancer?

Yes ☐ No ☐

5. Do you have a have a parent or sibling with breast cancer?

Yes ☐ No ☐

6. Why are you attending the breast clinic today?

Investigation of a breast symptom ☐ Breast Biopsy ☐
Mammogram for history of breast cancer ☐ Clinic Follow-up ☐
Mammogram for family history ☐ Other – Please Specify ___________

7. How would you describe your interest in AI in general?

Strong Interest ☐ Some Interest ☐ No Interest ☐ Don't know ☐

8. The use of AI in healthcare is a good idea.

Strongly agree ☐ Agree ☐ Neither agree or disagree ☐ Disagree ☐ Strongly Disagree ☐

9. In the future would you agree to an AI tool reading your mammogram in addition to a doctor who reports images (radiologist)? (Mammogram is read by AI and a single human reader)

Strongly agree ☐ Agree ☐ Neither agree or disagree ☐ Disagree ☐ Strongly Disagree ☐

10. In the future would you approve for an AI tool to be the only reader of your mammogram? (Only the AI tool reads your mammogram - No human reader)

Strongly agree ☐ Agree ☐ Neither agree or disagree ☐ Disagree ☐ Strongly Disagree ☐

11. How do you feel about the following statement?
I think replacement of breast radiologists by AI could happen in the future.

Strongly agree ☐ Agree ☐ Neither agree or disagree ☐ Disagree ☐ Strongly Disagree ☐

12. How do you feel about the following statement?
Even if AI tools are *more efficient and quicker* at reading mammograms, I still prefer a human radiologist to read my mammogram.

Strongly agree ☐ Agree ☐ Neither agree or disagree ☐ Disagree ☐ Strongly Disagree ☐

13. Even if AI tools are *more accurate* at reading mammograms, I still prefer a human radiologist to read my mammogram.

Strongly agree ☐ Agree ☐ Neither agree or disagree ☐ Disagree ☐ Strongly Disagree ☐

14. In your opinion if a mammogram is read by a radiologist AND an AI tool, and an *incorrect result* is given to the patient, who is at fault?

Developer of AI tool ☐ Human radiologist ☐ Both ☐ Neither ☐

15. How do you feel about the following statement?
If my mammogram images were fully anonymised I would feel comfortable sharing my images with other hospitals or AI companies to help develop and improve AI tools for future detection of breast cancer.

Strongly agree ☐ Agree ☐ Neither agree or disagree ☐ Disagree ☐ Strongly Disagree ☐

Thank you for taking the time to complete this survey.

Supplementary figure 1 –

Questionnaire on patient perceptions on the use of AI in breast clinic

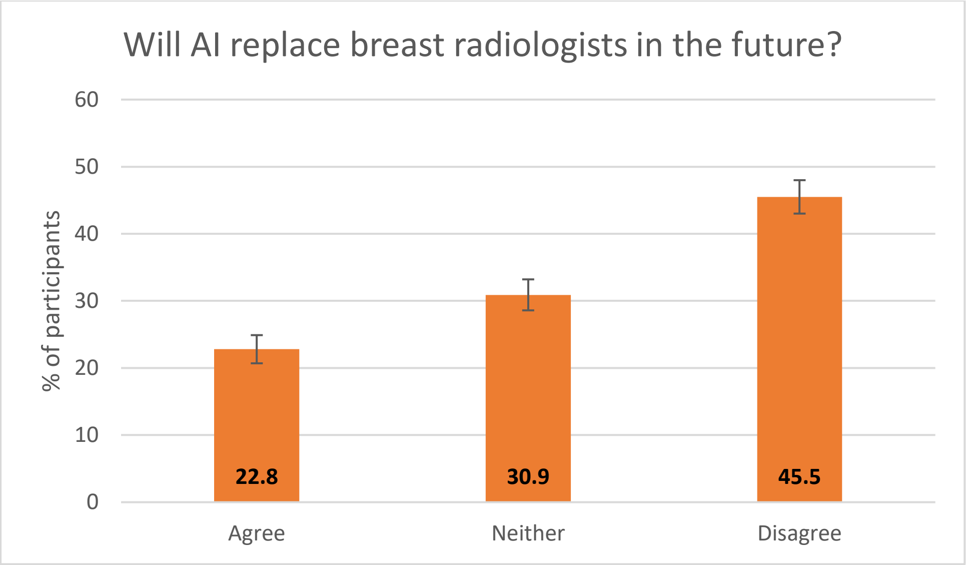

Supplementary Figure 2. Responses to question asking participants whether they feel AI will replace breast radiologists in the future. The y axis represents percentage of participants with the error bars representing 95% confidence intervals.

Supplemental Table 1. Multinomial logistic regression on the question asking participants if they prefer a radiologist even if AI is more accurate

**Prefer a radiologist even if AI more accurate – agree**	**Relative Risk (RRR)**	**P value**	**95% confidence interval**
**Age**			
<40 years	1 (base)		
40-49 years	1.25	0.205	0.89-1.75
50-69 years	1.60	0.013	1.12-2.32
>70 years	1.84	0.038	1.03-3.28
**Education level**			
Leaving certificate & below	1 (base)		
PLC/Bachelors & above	1.02	0.866	0.78-1.35
**History of breast cancer**			
Family history of breast cancer	0.92	0.608	0.69-1.25

Personal history of breast cancer	1.29	0.127	0.93-1.79
**Interest in AI**			
No interest	1 (base)		
Some interest	0.86	0.358	0.62-1.19
Strong interest	1.17	0.506	0.74-1.85
Don’t know	0.88	0.542	0.58-1.33
**Neither agree or disagree**	**Base outcome**		
**Prefer a radiologist even if AI more accurate – disagree**	**Relative risk (RRR)**	**P value**	**95% confidence interval**
**Age**			
<40 years	1 (base)		
40 – 49 years	0.84	0.553	0.48 – 1.48
50 – 69 years	1.25	0.464	0.69 – 2.28

>70 years	0.37	0.217	0.08 – 1.78
**Education level**			
Leaving certificate & below	1 (base)		
PLC/Bachelors & above	3.25	<0.001	1.84 – 5.76
**History of breast cancer**			
Family history of breast cancer	1.34	0.224	0.84 – 2.15
Personal history of breast cancer	1.18	0.565	0.68 – 2.04
**Interest in AI**			
No interest	1 (base)		
Some interest	0.91	0.752	0.52 – 1.61
Strong interest	2.14	0.031	1.07 – 4.28
Don’t know	0.48	0.124	0.19 – 1.22

Supplemental Table 2. Distribution of all responses to all questions in the questionnaire

**Age**	**<30**	**30-39**	**40-49**	**50-59**	**60-69**	**>70**	**Unknown**	
	50 (3%)	233 (15%)	549 (36%)	402 (26%)	163 (11%)	135 (9%)	2 (0.1%)	
**Ethnicity**	**White Irish**	**Irish traveller**	**Any other white background**	**Black, African**	**Any other black background**	**Asian, Chinese**	**Any other Asian background**	**Other**
	1267 (83%)	6 (0.4%)	166 (11%)	26 (2%)	0	27 (2%)	20 (1%)	22 (1.4%)
**Education**	**Junior certificate or below**	**Leaving certificate or equivalent**	**PLC course**	**Bachelor's degree**	**Master's or higher degree**	**Unknown**		
	184 (12%)	386 (25%)	224 (15%)	411 (27%)	270 (18%)	59 (4%)		
**Previous breast cancer diagnosis**	**Yes**	**No**	**Unknown**					
	372 (24%)	1154 (75%)	8 (0.5%)					
**Family history of breast cancer**	**Yes**	**No**	**Unknown**					
	367 (24%)	1145 (75%)	22 (1%)					
**Reason for breast clinic attendance**	**Investigation of a breast symptom**	**Mammogram for history of breast ca**	**Mammogram for family history**	**Breast biopsy**	**Clinic follow up**	**Unknown**		
	770 (50%)	294 (19%)	201 (13%)	7 (0.5%)	236 (15%)	26 (2%)		
**Interest in AI**	**No interest**	**Some interest**	**Strong interest**	**Don't know**				

	331 (22%)	732 (48%)	211 (14%)	235 (15%)	
**Use of AI in healthcare is a good idea**	**Agree**	**Neither**	**Disagree**	**Unknown**	
	711 (46%)	673 (44%)	113 (7%)	37 (2%)	
**Mammogram read by AI & radiologist**	**Agree**	**Neither**	**Disagree**	**Unknown**	
	935 (61%)	392 (26%)	198 (13%)	9 (1%)	
**Mammogram read by AI only**	**Agree**	**Neither**	**Disagree**	**Unknown**	
	160 (10%)	339 (22%)	1026 (67%)	9 (1%)	
**AI could replace radiologists**	**Agree**	**Neither**	**Disagree**	**Unknown**	
	350 (23%)	474 (31%)	698 (46%)	12 (1%)	
**Even if AI more efficient, still prefer a radiologist**	**Agree**	**Neither**	**Disagree**	**Unknown**	
	1156 (75%)	286 (19%)	72 (5%)	20 (1%)	
**Even if AI more accurate, still prefer a radiologist**	**Agree**	**Neither**	**Disagree**	**Unknown**	
	1014 (66%)	364 (24%)	125 (8%)	31 (2%)	
**Who is accountable for an incorrect result**	**AI developer**	**Radiologist**	**Both**	**Neither**	**Unknown**
	91 (6%)	154 (10%)	1130 (74%)	91 (6%)	68 (4%)
**Willing to share images for AI research**	**Agree**	**Neither**	**Disagree**	**Unknown**	
	1083 (71%)	235 (15%)	154 (10%)	28 (2%)	
